# NeuroToolKit Data Hackathon: advancing data collaboration in Alzheimer's disease

**DOI:** 10.3389/fnins.2024.1339742

**Published:** 2024-06-27

**Authors:** Craig Ritchie, Kaj Blennow, Juan Domingo Gispert, Sterling Johnson, Ingrid van Maurik, Lisa Vermunt, Marc Suárez-Calvet, Caitlin P. McHugh, Matthew H. S. Clement, Alexandra Anastasiu, Eugen Rosenfeld, Oana Cosma, Chad A. Logan, Frances-Catherine Quevenco, Mariana Castro Dias, Margherita Carboni

**Affiliations:** ^1^Scottish Brain Sciences, Edinburgh, United Kingdom; ^2^Edinburgh Dementia Prevention and Centre for Clinical Brain Sciences, Edinburgh Medical School, University of Edinburgh, Edinburgh, United Kingdom; ^3^Department of Psychiatry and Neurochemistry, Institute of Neuroscience and Physiology, University of Gothenburg, Mölndal, Sweden; ^4^Barcelonaβeta Brain Research Center (BBRC), Pasqual Maragall Foundation, Barcelona, Spain; ^5^Hospital del Mar Medical Research Institute (IMIM), Barcelona, Spain; ^6^Universitat Pompeu Fabra, Barcelona, Spain; ^7^Centro de Investigación Biomédica en Red Bioingeniería, Biomateriales y Nanomedicina (CIBER-BBN), Madrid, Spain; ^8^School of Medicine and Public Health, University of Wisconsin-Madison, Madison, WI, United States; ^9^Epidemiology and Data Science, Amsterdam UMC, Vrije Universiteit Amsterdam, Amsterdam, Netherlands; ^10^Neurology, Alzheimer Center Amsterdam, Amsterdam UMC, Vrije Universiteit Amsterdam, Amsterdam, Netherlands; ^11^Amsterdam Neuroscience, Neurodegeneration, Amsterdam, Netherlands; ^12^Neurochemisty Laboratory, Clinical Chemistry, Amsterdam UMC, Vrije Universiteit Amsterdam, Amsterdam, Netherlands; ^13^Centro de Investigación Biomédica en Red de Fragilidad y Envejecimiento Saludable (CIBERFES), Madrid, Spain; ^14^Servei de Neurología, Hospital del Mar, Barcelona, Spain; ^15^Alzheimer's Disease Data Initiative, Kirkland, WA, United States; ^16^Nagarro, Cluj-Napoca, Romania; ^17^Roche Diagnostics GmbH, PHCS Biostatistics & Data Management, Penzberg, Germany; ^18^Roche Diagnostics International Ltd, Rotkreuz, Switzerland

**Keywords:** Alzheimer's disease, cloud-based workbench, collaborative research, NTKApp, online community, virtual platform

## 1 Introduction

Across the healthcare industry, biomarkers (e.g., blood or cerebrospinal fluid [CSF], imaging, and digital markers) are widely used as screening or diagnostic tools, for therapeutic drug monitoring, or to guide clinical decision-making (Gromova et al., 2020). There is considerable interest in identifying and validating biomarkers for neurodegenerative disease research and clinical trials, as well as routine use (i.e., diagnostic, prognostic, and treatment selection), including for Alzheimer's disease (AD) (Blennow and Zetterberg, 2018; Hampel et al., 2018; Zetterberg and Bendlin, 2021). However, reviewing the vast number of publications to identify and qualify viable biomarker candidates requires both assay compatibility and statistical methods to facilitate the communication of knowledge, data integration, and visualization.

Collaborative research environments allow researchers from various communities and geographies to compare statistical analysis and interpret and contextualize results, while enabling data privacy/ownership, secure storage, and controlled access. This allows the development of novel insights from available data, and the replicability of knowledge and results, while ensuring conformity to regulatory, privacy, and intellectual property requirements. In addition to maintaining data privacy/ownership, there is an urgent need to enhance resources and tools supporting the findability, accessibility, interoperability, and reusability of available data (i.e., the FAIR Data Principles) (Wilkinson et al., 2016). The provision of cloud-based, global, collaborative platforms provides users with the opportunity to identify key questions and formulate queries, with the aim of obtaining rapid and insightful solutions. The use of standardized statistical analysis tools allows knowledge sharing by maximizing comparability and interpretability of results.

One such tool is the NeuroToolKit Application (NTKApp; https://www.alzheimersdata.org/ntk/ntkapp-platform),^1^ developed by Roche Diagnostics during 2021 and supported by the Alzheimer's Disease Data Initiative, a non-profit organization. The NTKApp allows standardization of statistical analysis and creation of statistical analysis modules, allowing comparability and reproducibility of results and thus generation of replicable scientific evidence. The results can be used to generate insights and inform the neurology field of the potential clinical utility of biomarkers for *in vitro* diagnostic tests and clinical trials and practice.

The prototype of the NTKApp was publicly beta-tested in July 2022 and is now available via the AD Workbench, Alzheimer's Disease Data Initiative's secure, cloud-based, collaborative data platform, and research environment. The availability of the NTKApp and AD Workbench offers an innovative solution to collaborative data sharing and interpretation. Here, we report on the NTKApp beta-testing phase and how this tool could advance neurology research, particularly in AD.

## 2 Methods

The Alzheimer's Disease Data Initiative NeuroToolKit (NTK) Data Hackathon virtual event^2^ took place from July 1–17, 2022. Global researchers, biostatisticians, data scientists, and clinicians were invited to join in self-selected teams of 2–4 (*n* = 45) with the aim of investigating the clinical utility of different cerebrospinal fluid biomarkers in AD through a set of challenges (i.e., proposed scientific questions).

Participants were given access to the AD Workbench, where they could use the NTKApp and other statistical analysis tools (i.e., R and Python) to explore permissioned datasets from the European Prevention of Alzheimer's Dementia Consortium (Ritchie et al., 2016). The European Prevention of Alzheimer's Dementia datasets comprise data on outcomes associated with early-stage neurodegenerative disease, including socio-demographic data, cognitive assessment results neurodegenerative disease, cerebrospinal fluid markers, brain imaging, psychologic assessments, medical history, physical examinations, stress, sleep, quality of life, and life events. The AD Connect online community enabled participants to engage with team members and other participants and access resources including Hackathon information, the NTKApp user guide, technical help, and user forums. Participants could write posts to seek help or share experiences or useful materials.

The Hackathon challenges set by experts around common interests in AD and the available dataset were: (i) to explore and evaluate the amyloid, tau, neurodegeneration (ATN) framework biomarkers (Jack et al., 2018) and (ii) to explore the relationship between biomarkers and additional AD risk factors. To complete the Hackathon, teams performed exploratory data analysis, created reports, shared results, and submitted a short (3–5-min) video to present methodology, analyses, and findings.

The first challenge required teams to explore how the ATN criteria are associated with profiles that may include cognitive, functional, and neuropsychiatric features in high-risk individuals, and provide additional insights into how patients may be clustered into different risk profiles (Ebenau et al., 2020; Ingala et al., 2021). Teams evaluated and sought to improve the ATN biomarker definitions, and compared them with the definitions used in published studies. The second challenge (optional) required teams to identify factors or variables that best differentiated biomarker amyloid-positive from amyloid-negative individuals in a population of older, cognitively normal adults, and to identify different ways to classify this population based on risk factors for cognitive impairment. Additional tasks to further explore the dataset and stimulate ideas that might be useful in clinical context included defining different assay cut-offs as compared with Elecsys^®^ cerebrospinal fluid immunoassay (Roche Diagnostics International Ltd, Rotkreuz, Switzerland) cut-offs, and evaluating the potential clinical use of β-amyloid (1–42), phosphorylated-tau, and total-tau in older, cognitively normal adults.

## 3 Results

Of the 45 teams, 10 teams submitted videos, which were scored by six judges with expertise in the field (see text footnote^2^). There were four categories of assessment: (1) Patient Value and Clinical Impact; (2) Scientific Value; (3) Innovation; and (4) Technical. Each category had a winning team based upon the highest score, with each team able to win one category only. Teams were also scored on a Community Team Contribution category based on the value of each team's contributions to the AD Connect online community, including the number of posts and engagement received from other participants during the Hackathon. The winners were offered symbolic prizes (e.g., a drone).

**Figure 1:**
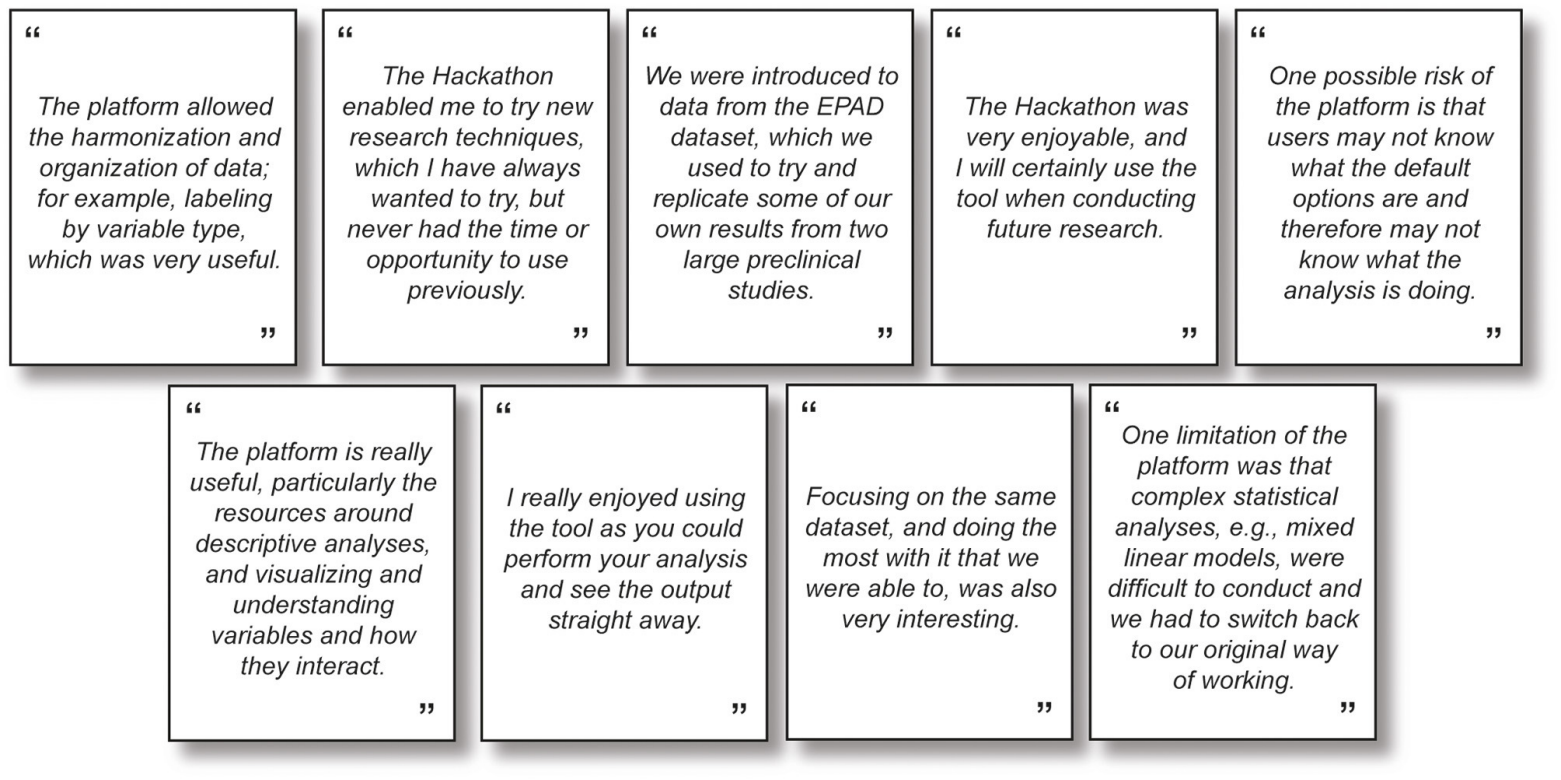
Advantages and limitations of the NTKApp from members of the winning teams (Team Barcelona Neuro Hacker, Spain, and Team UW Badgers, USA).

The Hackathon provided an opportunity for participants to build scripts maximizing their own analytical skills, contribute to AD research, and have fun, as evidenced by positive feedback (Figure 1). An example of results generated from the Hackathon is illustrated by one of the winning teams, who used the AD Workbench and European Prevention of Alzheimer's Dementia dataset to identify modifiable risk factors associated with ATN biomarkers (Roccati et al., 2024). Traumatic brain injury, obesity, and smoking were significantly associated with phosphorylated-tau; traumatic brain injury, obesity, smoking, depression, and physical inactivity were significantly associated with neurodegeneration (Supplementary Video 1; video from Team Wicking). This could be clinically useful to show patients their known biomarker profile alongside modifiable risk factors in an easy-to-interpret format to allow for lifestyle intervention to reduce their risk of AD. Supplementary Video 2 (video from Team MD Eagles) shows results generated by Team MD Eagles.

## 4 Discussion

The NTKApp is a collaborative analytics tool consisting of a suite of data harmonization and standardized analysis tools in three interlinked applications (apps) (Logan et al., 2023): (i) the NTKCuration app for standardization and harmonization of uploaded data; (ii) the NTKAnalysis app for data visualization, exploration, analysis, reporting, and collaborative interpretation of results; and (iii) the NTKMeta-Analysis app for systematic assessment of multiple analyses, to evaluate reproducibility and strength of evidence, while maintaining data ownership and privacy. Tutorials are available on how to access the NTKApp and use the three interlinked apps (Supplementary Videos 3–6; NTKApp tutorials).

The NTKApp enables users to upload, curate, analyze, and compare biomarker datasets and results, and integrate these with a range of risk and other phenotypic data. The app offers a user-friendly tool to improve the way researchers collaborate and interpret neurologically relevant immunoassay data. The AD Workbench, which comprises tools such as the NTKApp, aids in breaking traditional silos in research, provides a forum for an exchange of ideas and research support, and helps to foster partnerships between academia and industry. It is anticipated that results shared from, and data analyses performed in, the NTKApp will help to inform the development of biomarkers for *in vitro* diagnostic tests, and to clarify their use in clinical trials and practice. The NTKApp was developed to explore the main clinical uses of biomarkers, namely screening, diagnosis, prediction and prognosis, and pharmacodynamic or efficacy monitoring. Screening and diagnosis could aid in detecting or confirming the presence of AD pathology, while biomarkers for prediction and prognosis will forecast the likely course of disease in untreated individuals and identify patients who are likely to progress faster. Biomarkers for pharmacodynamic or efficacy monitoring will indicate whether a biologic response to treatment has occurred, serve as surrogates for clinical efficacy endpoints, or distinguish between patients who may or may not respond to therapy.

Based on feedback received following the Hackathon, a scalable, modular first version of NTKApp was released exclusively via the AD Workbench in September 2022. Continuous updates will be implemented to expand its collaboration and analysis capabilities (e.g., additional biomarkers and diseases) based on user and partner engagement and feedback.

Two of the biggest obstacles to innovative biomarker research are limited accessibility to data and inconsistent, siloed information sources. The NTKApp aims to provide interoperability and a collaborative framework within an enormous database of standardized information, allowing users to upload and explore information, develop and validate hypotheses, and analyze and display findings for wider sharing. The NTKApp implements various aspects of the FAIR Data Principles (Wilkinson et al., 2016), which define characteristics that contemporary data resources and tools should exhibit to assist discovery and reuse by third parties. Online data resources and tools are considered democratic when provision is free, which is the case with the NTKApp and AD Workbench. This freedom and interoperability facilitates the undertaking of high-quality research globally, including more marginalized communities such as low- and middle-income countries. It is hoped that this tool, and others, will revolutionize healthcare research, leading to the development of clinically invaluable tools for AD diagnosis, prognosis, and treatment monitoring.

Collaborative research, particularly between institutions, is a challenge due to differences in data preparation methods, coding standards, and coding environments. Although the NTKApp provides a collaborative framework that enables researchers to share standardized methods, the app creators do not dictate which methods and standards should be used. In doing so, members of the research community are encouraged to develop the appropriate methods and standards for integration into the app for use in collaborative research and distribution to the research community as a whole. Thus, appropriate methods and standards can be developed and adapted over time to meet future needs.

The NTKApp has undergone substantial technical upgrades since the Hackathon based on user feedback. In the future, use of the NTKApp will be broadened to become disease and biomarker agnostic—a new name is currently under discussion.

## Author contributions

CR: Writing—original draft, Writing—review & editing. KB: Writing—original draft, Writing—review & editing. JG: Writing—original draft, Writing—review & editing. SJ: Writing—original draft, Writing—review & editing. IM: Writing—original draft, Writing—review & editing. LV: Writing—original draft, Writing—review & editing. MS-C: Writing—original draft, Writing—review & editing. CM: Writing—original draft, Writing—review & editing. MCl: Writing—original draft, Writing—review & editing. AA: Writing—original draft, Writing—review & editing. ER: Writing—original draft, Writing—review & editing. OC: Writing— original draft, Writing—review & editing. CL: Writing—original draft, Writing—review & editing. F-CQ: Writing—original draft, Writing—review & editing. MD: Writing—original draft, Writing—review & editing. MCa: Writing—original draft, Writing—review & editing.

## Footnotes

^1^Alzheimer's Disease Data Initiative (2022). *The NTKApp*. Available online at: https://www.alzheimersdata.org/ntk/ntkapp-platform (accessed April 24, 2024).

^2^Alzheimer's Disease Data Initiative (2022). *ADDI NeuroToolKit Data Hackathon*. Available online at: https://www.alzheimersdata.org/news/addi-neurotoolkit-data-hackathon (accessed April 24, 2024).
