# Supplementary material for: NeuroToolKit Data Hackathon: advancing data collaboration in Alzheimer's disease
Source: Front Neurosci. 2024 Jun 27;18:1339742. doi: 10.3389/fnins.2024.1339742 (PMC11238719; doi:10.3389/fnins.2024.1339742)

Supplementary Material

NeuroToolKit Data Hackathon: advancing data collaboration in Alzheimer's disease

Supplementary Video 1. Patient Value and Clinical Impact winning video (Team Wicking; Australia).

The video submitted by Team Wicking (Australia) who won the Patient Value and Clinical Impact category. Team Wicking used the NTKApp, AD Workbench, and EPAD dataset to identify modifiable risk factors associated with ATN biomarkers. They found TBI, obesity, and smoking were significantly associated with phosphorylated-tau; TBI, obesity, smoking, depression, and physical inactivity were significantly associated with neurodegeneration.

AD: Alzheimer's disease, ATN: Amyloid, tau, neurodegeneration, EPAD: European Prevention of Alzheimer's Dementia, NTKApp: NeuroToolKit application, TBI: Traumatic brain injury.

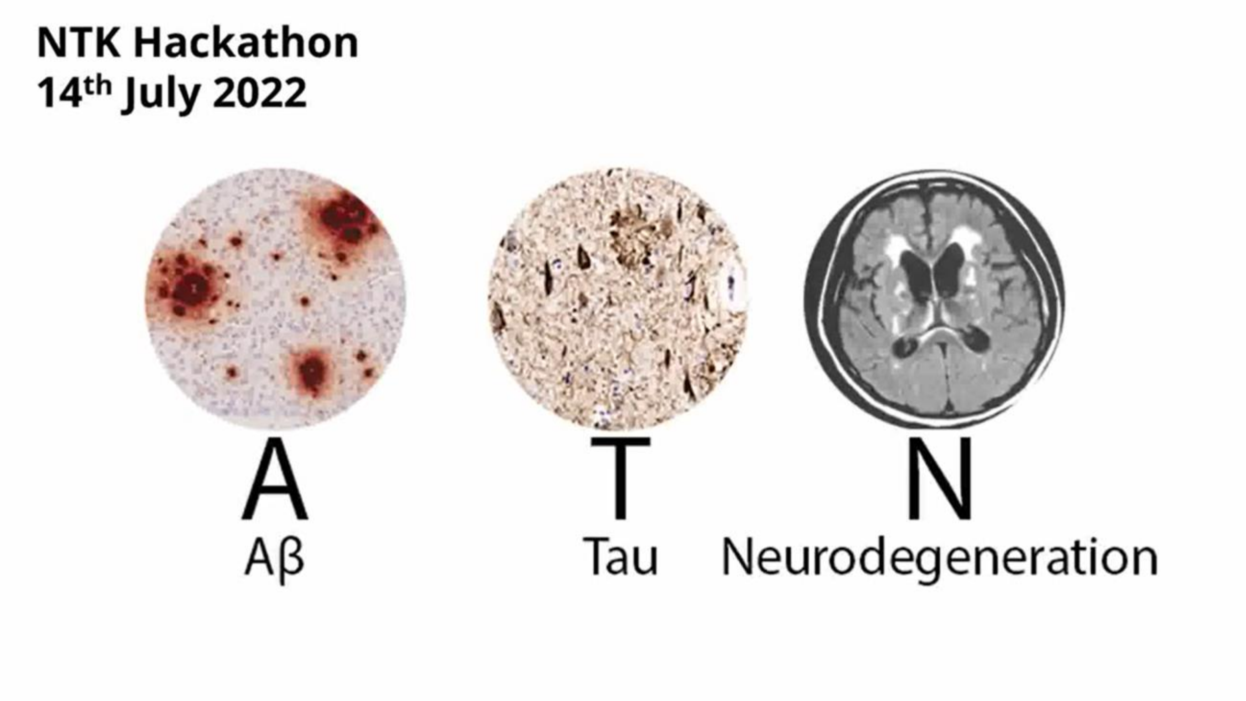

Supplementary Video 2. Technical Contribution winning video (Team MD Eagles; USA).

The video submitted by Team MD Eagles (USA) who won the Technical category. Team MD Eagles used the NTKApp, AD Workbench, and EPAD dataset to identify high-risk participants associated with the ATN biomarkers using a model-based clustering algorithm to capture the underlying structure of the data. Once high-risk individuals are identified, interventions could be provided to prevent or delay progression of AD.

AD: Alzheimer's disease, ATN: Amyloid, tau, neurodegeneration, EPAD: European Prevention of Alzheimer's Dementia, NTKApp: NeuroToolKit application.

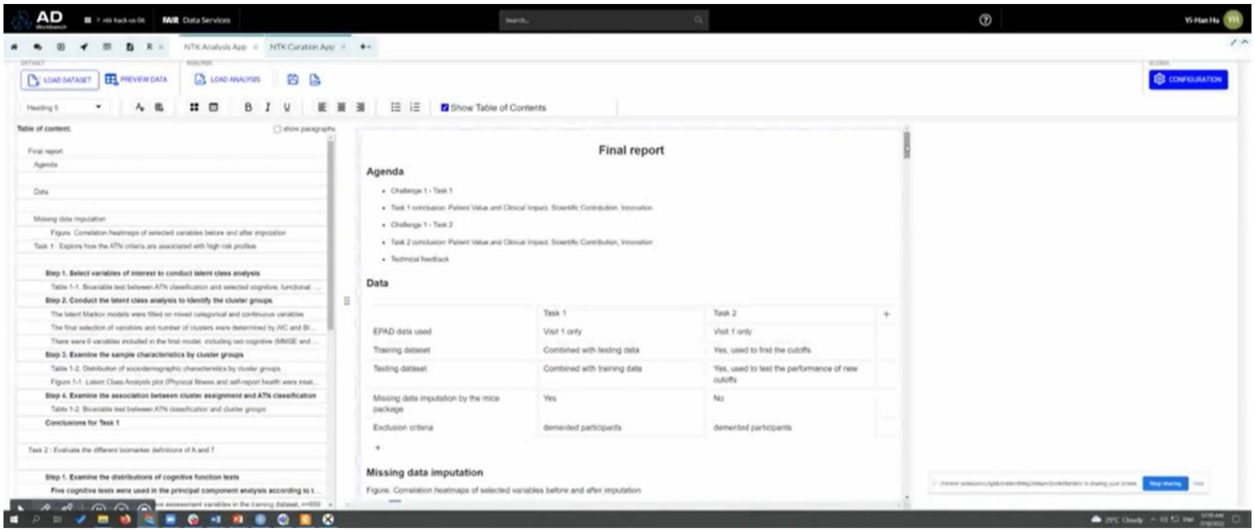

Supplementary Video 3. Accessing the NTKApp.

A step-by-step tutorial on how to access the NTKApp on the AD Workbench.

AD: Alzheimer’s disease, NTKApp: NeuroToolKit application.

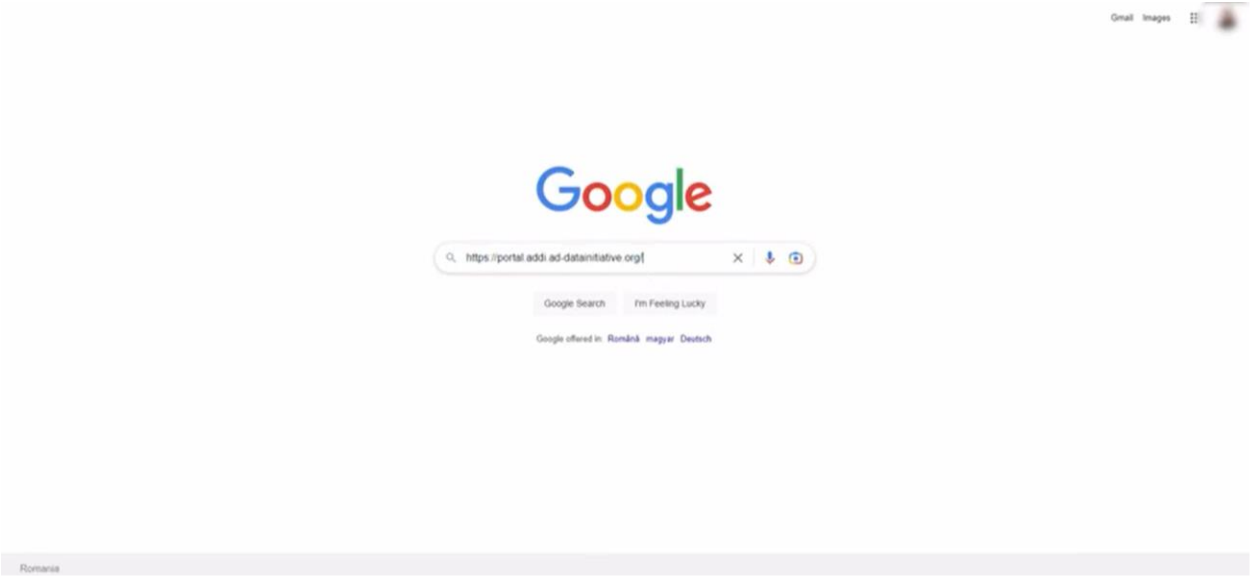

Supplementary Video 4. Using the NTKCuration app.

A step-by-step tutorial on how to use the NTKCuration tool, curate a dataset using standard and custom dictionaries, and prepare it for analysis.

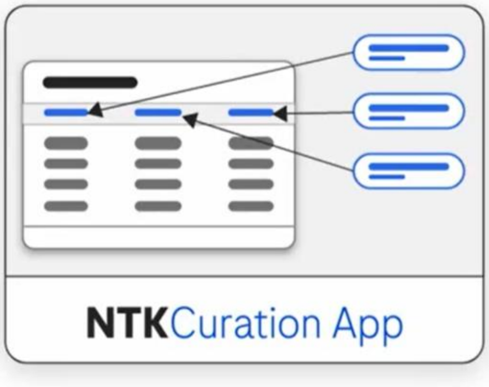

Supplementary Video 5. Using the NTKAnalysis app.

A tutorial how to use the NTKAnalysis tool and save a report in one interactive, publication-ready document. The NTKAnalysis tool bridges together R, RShiny, Python, and RMarkdown to create and execute statistical analyses.

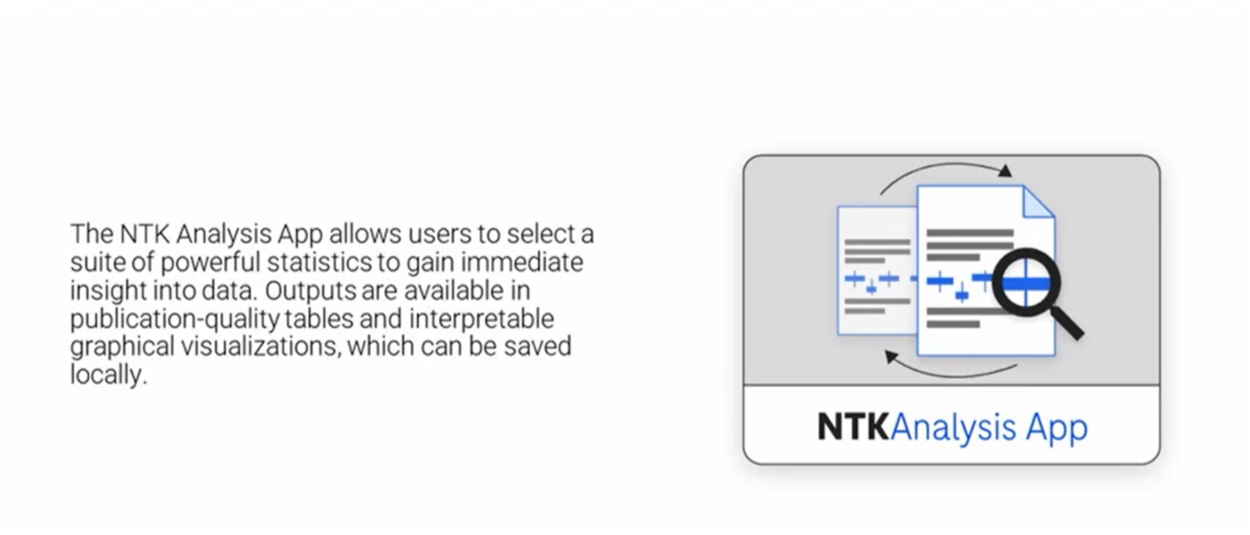

Supplementary Video 6. Using the NTKMeta-Analysis app.

A tutorial on how to use the NTKMeta-Analysis tool and compile multiple results into one interactive document. The NTKMeta-Analysis tool allows comparison of results generated by the NTKAnalysis tool across multiple studies or partners and produces aggregate point estimates.

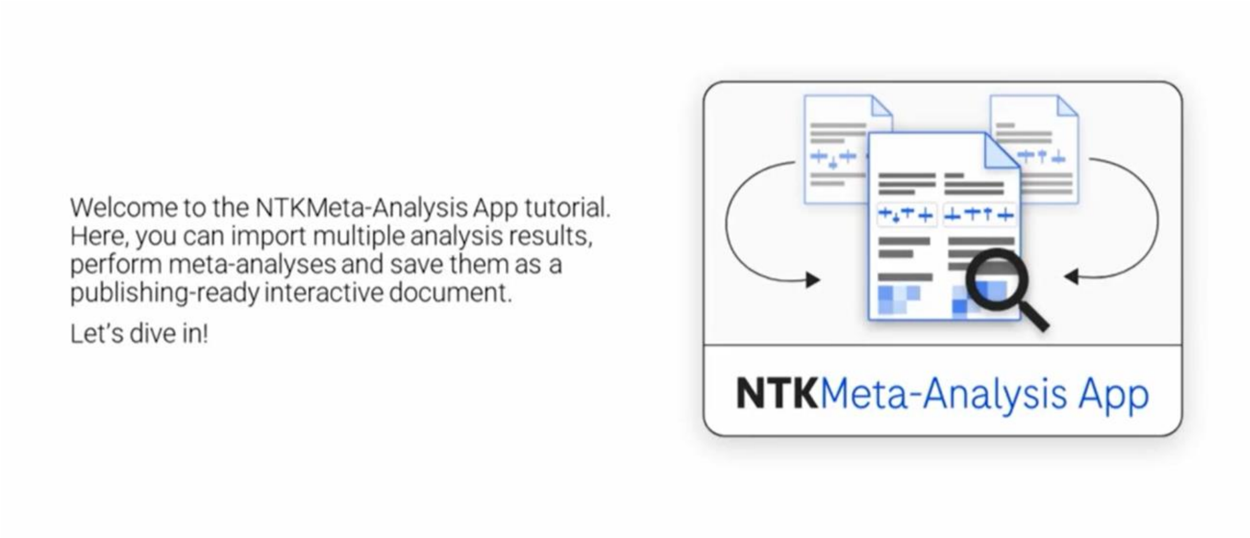

Supplement: Supplementary file 1 [file Data_Sheet_1.pdf]
